# Assessing the Effect of Malaria Exposure History on Tetanus Antibody Waning Rates Among Children in Jinja and Tororo Districts, Uganda

**DOI:** 10.1093/infdis/jiag255

**Published:** 2026-05-09

**Authors:** Alyssa N Sbarra, Will Stone, Kevin Tetteh, Isaac Ssewanyana, Kenneth Musinguzi, Isabel Rodríguez-Barraquer, Prasanna Jagannathan, Florian A Bach, Chris Drakeley, Saki Takahashi

**Affiliations:** Department of Epidemiology, Johns Hopkins Bloomberg School of Public Health, Baltimore, Maryland, USA; Department of Infection Biology, London School of Hygiene and Tropical Medicine, London, United Kingdom; Department of Infection Biology, London School of Hygiene and Tropical Medicine, London, United Kingdom; Infectious Diseases Research Collaboration, Kampala, Uganda; Infectious Diseases Research Collaboration, Kampala, Uganda; Division of HIV, Infectious Diseases and Global Medicine, Department of Medicine, University of California, SanFrancisco, San Francisco, California, USA; Department of Medicine, Stanford University, Stanford, California, USA; Department of Medicine, Stanford University, Stanford, California, USA; Department of Infection Biology, London School of Hygiene and Tropical Medicine, London, United Kingdom; Department of Epidemiology, Johns Hopkins Bloomberg School of Public Health, Baltimore, Maryland, USA

**Keywords:** tetanus, antibody, waning, vaccine, malaria

## Abstract

**Background:**

Malaria causes immune perturbations and affects immunogenicity to vaccines. It is unknown, however, how repeated malaria infections affect the duration of vaccine protection. This study assessed variability in tetanus antitoxin immunoglobulin G (IgG) antibody kinetics including by malaria exposure history at district and individual levels.

**Methods:**

We analyzed tetanus antitoxin IgG antibody concentrations in 3064 plasma samples from 2011 to 2017 in 597 children aged 6 months to 10 years residing in the Tororo (high malaria transmission) and Jinja (low transmission) districts in Uganda. Mixed-effects models were used to estimate antibody half-lives, and hazard models to estimate the risk of seroreversion.

**Results:**

Tetanus antitoxin IgG antibody concentrations were estimated to fall below protective levels by age 8.1 years [95% confidence interval (CI), 6.4–10.2 years]. Antibody half-lives were significantly longer in Jinja (15.8 [95% CI, 8.8–28.5] years) than in Tororo (2.8 [2.6–3.1] years). Hazard of seroreversion was significantly higher (2.7-fold [1.7–4.1-fold]) in Tororo than in Jinja.

**Conclusions:**

Tetanus antibodies waned rapidly. Children living in the district with high malaria transmission had faster waning and a higher risk of seroreversion than those in the district with low transmission, suggesting the need to determine if malaria endemicity should inform timing of tetanus booster delivery.

## Teaser

Tetanus immunity waned rapidly in Ugandan children, with antibody levels declining significantly without booster doses. The decay rate and hazard of seroreversion were greater in regions with higher malaria transmission, underscoring the need for booster doses, particularly in malaria-endemic regions.

**(See the Editorial Commentary by den Hartog and Rochford on pages e752–4.)**

The *Plasmodium falciparum* malaria parasite causes >200 million cases of malaria annually [1], mostly in highly endemic areas in sub-Saharan Africa. A malaria infection can result in a spectrum of clinical outcomes, including uncomplicated symptomatic malaria (ie, parasitemia with fever), asymptomatic infection (ie, parasitemia without fever), and rare but severe manifestations (eg, cerebral malaria or death). Malaria infections result in large antigenic loads in the blood and perturb the immunologic milieu [2–5]. These perturbations shape both immune response to *P. falciparum* and also other pathogens [6], such as exacerbating outcomes in coinfections with Epstein-Barr virus [7, 8] or nontyphoidal *Salmonella* [9].

Malaria also has bystander effects on host immune responses to childhood vaccine-preventable diseases (VPDs) starting early in life, with in utero malaria exposure associated with lower maternal placental antibody transfer [10, 11]. Furthermore, malaria parasitemia at the time of immunizations has been linked to poorer immunogenicity [12, 13]. While mechanisms underlying this reduced immunogenicity are not yet well defined, several explanations have been proposed, including immune imprinting yielding subsequent impaired ability to establish vaccine responses to VPD antigens [14]. Importantly, potential effects of recurrent malaria infections on the durability of VPD protection after vaccination—the premise of this study—are also poorly understood.

Tetanus, a VPD that also causes substantial disease and deaths [15], is an acute bacterial infection and has a high case fatality rate if untreated. Maternal and neonatal tetanus is an elimination target. Globally, immunization programs recommend administration of tetanus-containing vaccines in a 3-dose primary series given to infants before age 6 months. Because antibody-based correlates of protection have been established for tetanus [16], seronegativity is used as a proxy for susceptibility. Tetanus antibodies, and thus immunity, only follow vaccination (ie, not infection) and wane as children age [17]. Therefore, the World Health Organization currently recommends that 3 booster doses be administered following the primary vaccination series, at the ages of 12–23 months, 4–7 years, and 9–15 years [17]. Yet currently, many countries, including Uganda [18], administer only a tetanus-toxoid booster during routine antenatal care visits following the initial primary series delivered during infancy.

While tetanus antitoxin immunoglobulin G (IgG) antibody waning has been well described, it is unknown how tetanus antitoxin IgG antibody kinetics and durability of protection are affected by cumulative malaria infections experienced after vaccination. A key challenge to unraveling the relationship between malaria histories and tetanus susceptibility is that both vary between individuals and are independently expected to change over time. Thus, assessing this potential relationship requires longitudinal evaluation within the same individuals.

In the current study, we leveraged a well-characterized malaria cohort in 2 districts in Uganda with differing levels of malaria transmission [19–21], providing an exceptional example to study effects of recurrent malaria infections on tetanus immunity. We used serologic assays and statistical models to assess tetanus antitoxin IgG antibody kinetics and potential interactions with malaria exposure history.

## METHODS

This work leverages an extensive longitudinal cohort study of malaria in Uganda, which has been described extensively elsewhere [19, 21–23]. Briefly, this study (PRISM-1) was a population-based cohort of 1421 residents from 331 households followed up from 2011 to 2017. Within each household, all children between 6 months and 10 years of age were enrolled, along with a primary caretaker. In this analysis, we analyzed data from study participants from 2 of the 3 sites—Walukuba subcounty (Jinja district) and Nagongera subcounty (Tororo district)—who were aged between 6 months and 10 years during any part of the study period: [22]. Tororo is a rural district with highly endemic malaria transmission (in 2011–2013, median household annual entomologic inoculation rate (aEIR), 51 infectious bites per year; median incidence of symptomatic malaria, 2.6 episodes per person-year), and Jinja is an urban district with lower transmission (in 2011–2013, median household aEIR, 2.1; median incidence of symptomatic malaria, 0.6 episodes per person-year) [23].

Study participants were seen routinely every 12 weeks (ie, active surveillance), when they were screened for *P. falciparum* parasitemia by means of both microscopy and loop-mediated isothermal amplification, clinical symptoms, and other measurements (eg, weight). Participants were also followed up in the study clinic, open 7 days a week, for all illnesses to maximize detection of symptomatic malaria (ie, passive surveillance). At any visit, participants received antimalarial treatment if diagnosed with symptomatic malaria (ie, Ugandan standard of care). Each participant was tested for hemogoblin AS (HbAS), the sickle cell genetic trait, which has known associations with malaria [24]. Biospecimens, including up to 1 mL of plasma, were collected at each study visit. All primary data from the cohort are available on the ClinEpiDB platform [25].

We analyzed tetanus antitoxin IgG antibody responses from 3069 observations from 597 individuals from the Tororo and Jinja districts; these samples were selected agnostic to the timing of malaria infections. Samples were tested at the London School of Hygiene and Tropical Medicine on 1 of 2 malaria Luminex multiplex serology panels [26, 27]. Tetanus toxoid was included on the panels as a positive control antigen, as it was expected that individuals would have a response from prior vaccination. However, given this original purpose, tetanus international standards were not tested with the samples. Instead, a dilution series of a pooled hyperimmune serum from adults in a malaria-endemic setting from Tanzania (called “CP3”) was run on each plate.

We converted tetanus median fluorescent intensities (MFIs) to internationally recognized units (ie, international units per milliliter) via a multistep process leveraging CP3 dilution curves (Supplementary Figure 1). Overall, the tetanus antitoxin MFI of each sample in our study is first converted to the unit of CP3 concentrations. Then, since tetanus antitoxin IgG antibody concentrations (in international units per milliliter) of CP3 are known, these CP3 concentrations per sample are used to interpolate tetanus antitoxin IgG antibody concentrations on the scale of international units per milliliter. Specifically, we first converted tetanus antitoxin MFIs to values on the scale of CP3 concentration via a 4-point logistic model [28] (Supplementary Figure 2). We then compared tetanus antitoxin MFIs to international units per milliliter from an additional study [29] that used a Luminex assay with the same CP3 dilution curve and tetanus toxoid antigen and had included international tetanus standards [30]; we defined this relationship using a log-log linear model. Next, we converted tetanus on the CP3 concentration scale from this other study to international units per milliliter via a log-log model. Finally, we used these models to convert tetanus values from this study on the CP3 concentration scale to international units per milliliter. This made it possible to define our samples on the internationally recognized scale and to determine the serostatus of each sample. We used the internationally recognized cutoff value of seroprotection (0.1 IU/mL) to define seropositivity [31].

We performed statistical analyses on the final longitudinal dataset among children aged 6 months to 10 years among all samples that passed quality control (ie, with observed tetanus antitoxin MFI of ≥10 units, all 1650 samples measured from Jinja and 1414 of 1419 samples measured from Tororo).

We first assessed seroprevalence by age and sex by assessing overall rates of tetanus antitoxin IgG antibody decay across first observations for each individual. Then, using all available longitudinal data, we fit log-linear and log-log (ie, power) decay models with individual-level random intercepts from both study sites. Log-linear decay models yielded superior fits via an assessment of the Akaike information criterion and log-likelihoods (Supplementary Table 1). We then computed tetanus antitoxin IgG antibody concentration half-life from log-linear decay models, such that


$$\log\;(concentration)=\beta_{0}+\beta_{age}*age+id_{RE}\;,$$

where concentration is the tetanus antitoxin IgG antibody concentration (in international units per milliliter), β_0_ is an overall intercept, β_age_ is the regression coefficient for age, age is the age of the individual at the time of a given sample, and id_RE_ is a random intercept for each individual. We defined antibody concentration half-life as the time required for antibody concentrations to decrease by half, calculated from the decay rate (ie, β_age_).

Then, also using all available longitudinal data, we computed tetanus antitoxin IgG antibody concentration half-life from log-linear decay models by district, such that


$$\mathrm{Log}(\mathrm{concentration})=\beta_{0}+\beta_{\mathrm{age}}(\mathrm{age}\times \mathrm{district})+id_{\mathrm{RE}}\;,\;$$

where now we have an interaction between age and district. We used the delta method to estimate the 95% confidence interval (CI) of antibody concentration half-lives. We also computed the estimated age at seroreversion (ie, the age at which concentrations drop below the seroprotective threshold), using a Cox proportional hazard model for interval-censored data by district, such that


$$h_{i}(\mathrm{age})=h_{0}(\mathrm{age})e^{\beta_{district}*district}\;,\;$$

Where *h_i_* (age) is the hazard of seroreversion at a given age, *h*_0_ (age) is the baseline hazard function, and $e^{\beta_{district}*district}$ is the district-specific effect on the hazard.

We defined malaria exposure history in multiple ways: ecologically (by district), clinically (by parasitemia and malaria incidence), and entomologically (by aEIR). We assessed malaria exposure ecologically by comparing between the high (Tororo) versus low (Jinja) malaria transmission districts. Due to the combination of routine and nonroutine study visits that encompassed individuals’ malaria exposure histories, we further defined individual-level malaria exposure in multiple ways: (1) the proportion of months participants were enrolled in the study with any positive parasitemia, (2) the incidence of symptomatic (ie, febrile) malaria episodes per person-year, (3) the average individual hazard of malaria infection [23], and (4) household aEIR [23]. We also computed stunting status for all children and wasting status for those <5 years old at each study visit using information collected on height and weight. We compared antibody waning by nutritional status and HbAS genotype to consider other individual-level characteristics related to malaria exposure.

Models of additional malaria exposure were constructed, such that


$$\log\;(concentration)=\beta_{0}+\beta_{age}(age\times exposure\;group)+id_{RE}\;,$$

where district has been replaced by exposure group. Individual-level vaccination history data was not available, but tetanus-containing vaccine is administered at 6, 10, and 14 weeks of age in Uganda [32] with third-dose coverage reaching 82% in 2011% and 94% in 2017 [33]. While we cannot exhaustively exclude a chance of booster vaccination, we assume that these events are unlikely, especially as these children have not yet reached childbearing age (ie, attending antenatal care clinics).

Analyses were conducted in the R computing environment [34] (4.3.2). All computer code is publicly available on GitHub (https://github.com/alyssasbarra/prism_tetanus).

## RESULTS

We analyzed a final dataset of 3064 samples (range, 1–11 samples per individual; median, 5 samples per individual) from 597 individuals aged 6 months to 10 years from the Tororo and Jinja districts (Table 1). Overall, tetanus antitoxin IgG antibody concentrations and seroprevalence declined as age increased (Figure 1*A*), as also observed in predictions from fitted log-linear models. The observed seroprevalence among 6–11-month-olds was 87.5% (n = 48). By age 4 years, <75% of individuals were seropositive, and by age 8 years, 50% were seropositive. Overall, the tetanus antitoxin IgG antibody concentration half-life was estimated to be 3.9 years (95% CI, 3.5–4.5 years); the estimated age of seroreversion was 8.1 years (6.4–0.2 years). To visualize antibody concentrations without influence of individual-level variation across time, we also selected only the first sample that was tested from each individual and examined empirical distributions by age (Figure 1*B*); we again observed that antibody concentration distributions by age generally shifted left (ie, had lower values). Among these samples, there was no observed difference in seroprevalence among all ages by sex (Figure 2; *P* = .34, χ^2^); there were no significant differences in seroprevalence among all ages by sex in neither Jinja (*P* = .57) or Tororo (*P* = .33) districts.

**Figure 1.**
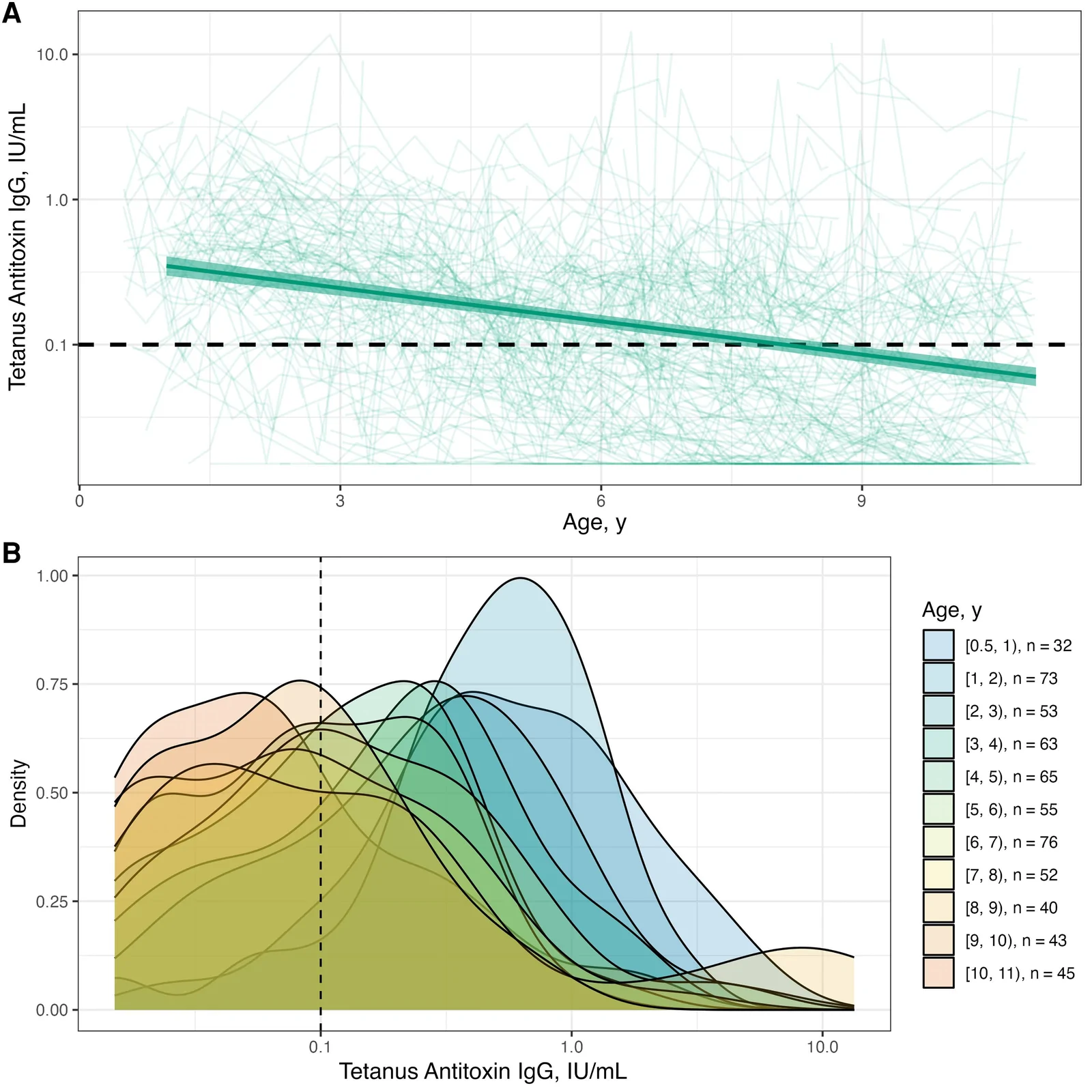
Tetanus antitoxin immunoglobulin G (IgG) antibody concentrations by age. *A*, Using all longitudinal samples from each individual, tetanus antitoxin IgG antibody concentrations decline by age; log-linear model predictions are shown as a dark green line, with 95% confidence intervals displayed as a lighter ribbon. *B*, Using the first tested sample from each individual, means from distributions of antibody concentrations by age also shifted left (ie, had lower values). Dashed horizonal (*A*) and vertical (*B*) lines indicate the seroprotective threshold of 0.1 IU/mL.

**Figure 2.**
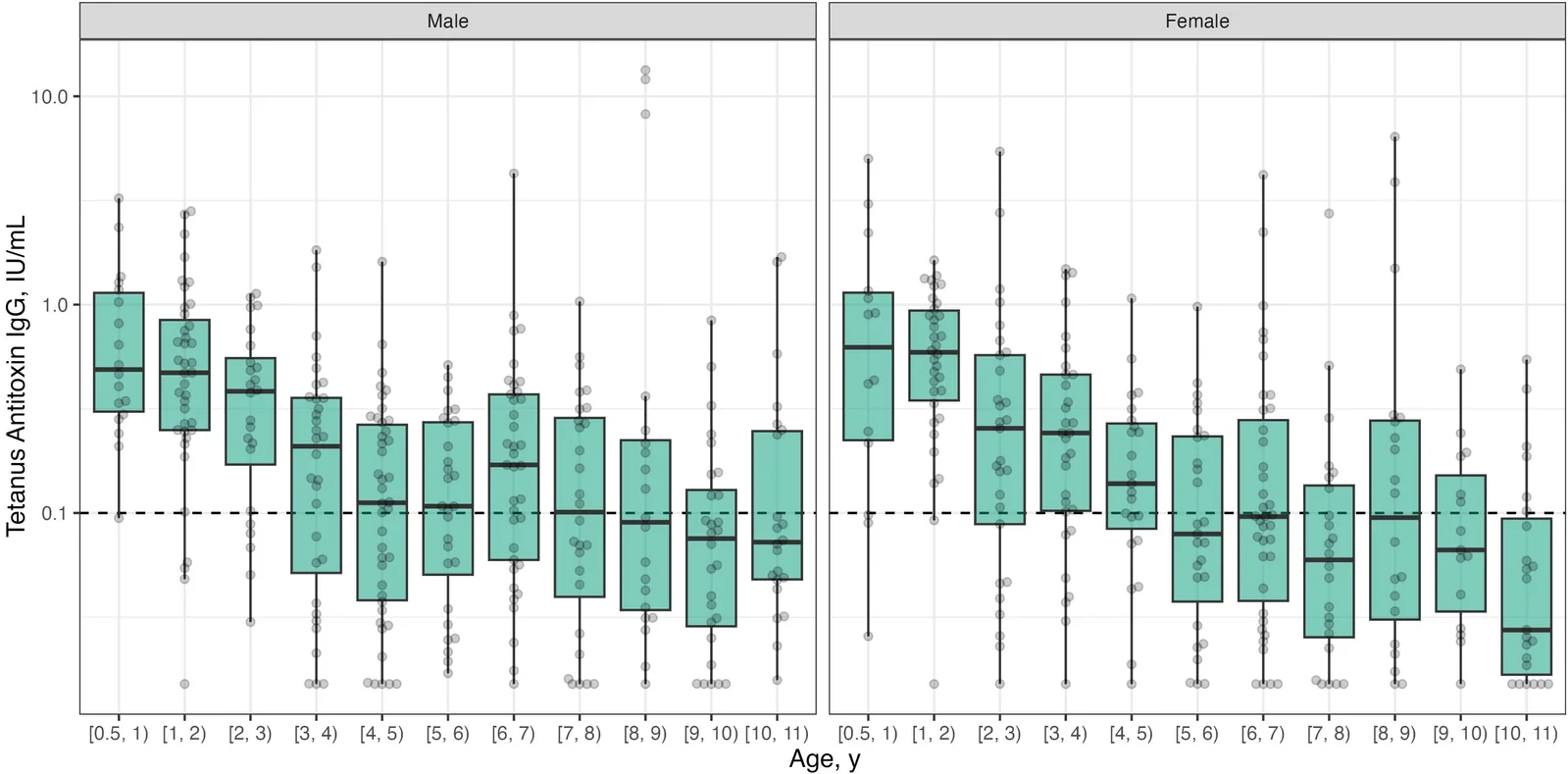
Box plots of tetanus antitoxin immunoglobulin G (IgG) antibody concentrations by age and sex at time of first observation for each individual. Dashed horizonal line indicates the seroprotective threshold of 0.1 IU/mL.

**Table 1.** Demographic and Malaria Exposure Features Among Children by Study Site

Feature	Children, No. (%)^a^	
	Jinja (n = 271)	Tororo (n = 326)
Females, no. (%)	129 (47.6)	151 (46.3)
Total analyzed samples, no.	1650	1414
No. of tetanus antitoxin IgG antibody observations per individual (%)		
1	22 (8.1)	57 (17.5)
2	16 (5.9)	22 (6.7)
>2	233 (86.0)	247 (75.8)
Age at earliest sample available, y		
Mean (SD)	4.94 (2.90)	5.65 (2.97)
Median (IQR)	4.75 (4.56)	5.80 (4.91)
Follow-up time, mean (SD), y	1.30 (0.69)	1.80 (1.12)
Annual individual-level febrile malaria incidence, mean (SD)	0.26 (0.61)	Pre-IRS period: 3.76 (3.42); post-IRS period: 0.98 (1.41)^b^
Proportion of months with parasitemia during study enrollment, mean (SD)	0.0682 (0.125)	Pre-IRS period: 0.466 (0.198); post-IRS period: 0.236 (0.243)^b^
Children who were ever stunted, no. (%)	31 (11.4)	42 (12.9)
Children <5 y old who ever experienced wasting, no. (%)	84 (49.1)	58 (33.9)
HbS genotype, no. (%)		
HbAA	208 (76.8)	209 (64.1)
HbAS	58 (21.4)	86 (26.4)
HbSS	3 (1.1)	1 (0.3)
Unknown	2 (0.7)	30 (9.2)

Abbreviations: HbAA, hemoglobin AA (ie, normal hemoglobin); HbAS, hemoglobin AS (ie, sickle cell genetic trait); HbS, hemoglobin type; HbSS, hemoglobin SS (ie, sickle cell disease); IgG, immunoglobulin G; IQR, interquartile range; IRS, indoor residual spraying.

^a^Data represent no. (%) of individuals unless otherwise specified.

^b^The pre-IRS and post-IRS periods were defined as before and after 1 January 2015; the IRS campaign took place in Tororo and not in Jinja.

We compared antibody decay across the 2 districts, which had different malaria transmission intensities (Figure 3). The half-life of tetanus antitoxin IgG antibody concentrations in Jinja (low transmission; 15.8 [95% CI, 8.8–28.5] years) was significantly longer than in Tororo (high transmission; 2.8 [2.6–3.1] years), suggesting faster rates of antibody decay in the district with higher malaria transmission. The estimated age at seroreversion was lower in Tororo (5.4 [95% CI, 4.5–6.5] years) than in Jinja (30.2 [15.4–59.3] years]). We also controlled for anthropometric status via both stunting and wasting to account for known lower immune function in malnourished children and the complex relationship between malaria and nutritional status [35]; the rate of tetanus antitoxin IgG antibody decay in Jinja was generally still significantly slower than in Tororo. However, there was no significant difference in the half-life of tetanus antitoxin IgG antibody concentrations among children within each district who were ever stunted or wasted within the study period (Supplementary Table 2).

**Figure 3.**
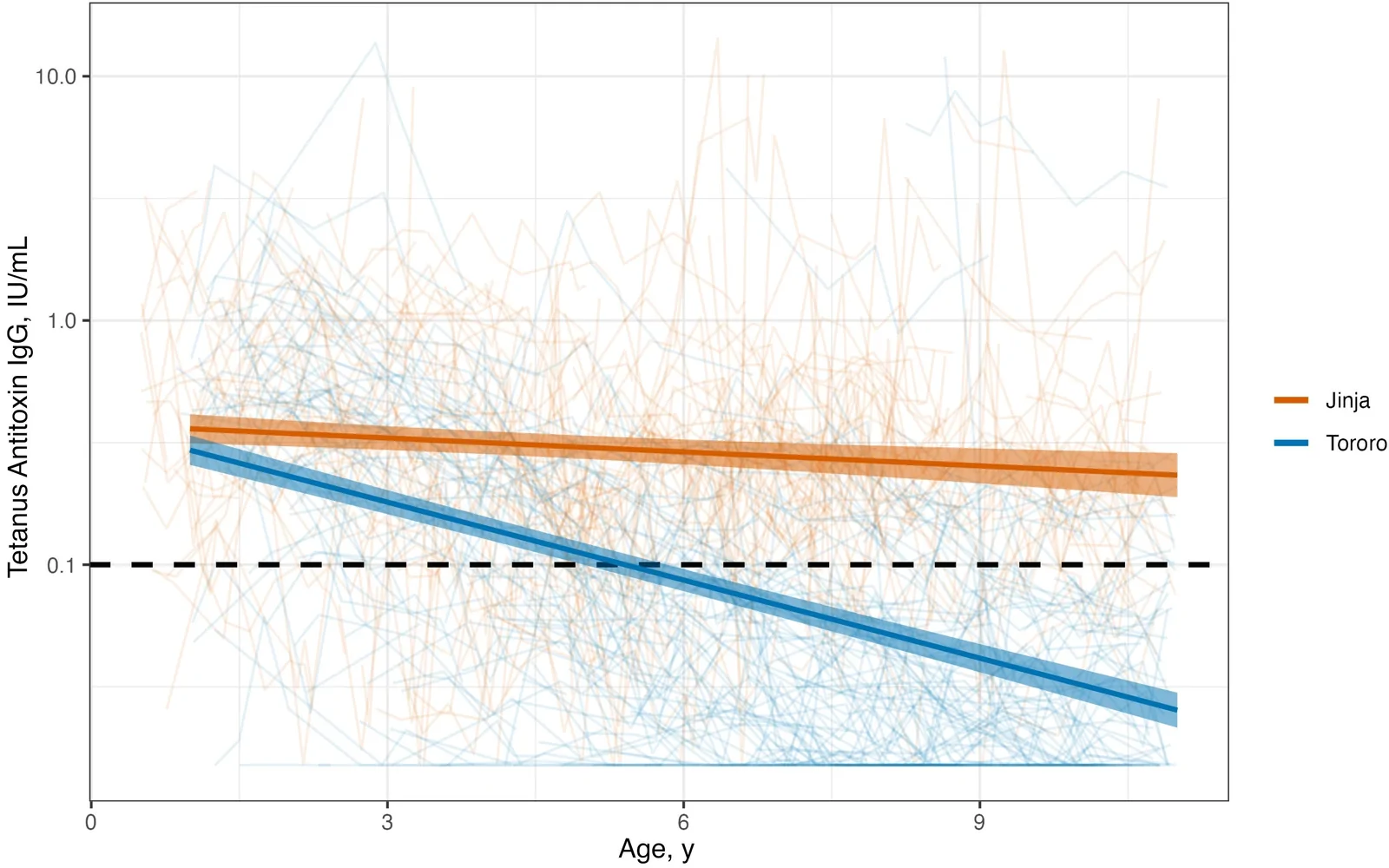
Tetanus antitoxin immunoglobulin G (IgG) antibody concentrations by age across study sites, with Jinja district in orange and Tororo district in blue. Log-linear model predictions by site shown in solid dark lines, with 95% confidence intervals displayed as lighter ribbons. Dashed horizonal line indicates the seroprotective threshold of 0.1 IU/mL.

We compared the risk of seroreversion across study sites. Among 320 children who were seropositive at the start of the study period (198 in Jinja and 122 in Tororo), 89 children (37 in Jinja and 52 in Tororo) seroreverted to concentrations <0.1 IU/mL during the study period. Using hazard models that adjust for age, we determined that there was a 2.7-fold (95% CI, 1.7–4.1-fold) higher hazard of seroreversion among children living in Tororo district who were seropositive at the start of the study period, relative to those living in Jinja district (*P* < .001; interval-censored Cox proportional hazard; Table 2 and Supplementary Figure 3). In a sensitivity analysis of 383 children who were ever seropositive during the study period (ie, to account for measurement error), we determined there was a 2.1-fold (95% CI, 1.5–2.9-fold) higher hazard of seroreversion among children living in Tororo relative to those living in Jinja (*P* = .005; Supplementary Table 3).

**Table 2.** Seroreversion by Study Site Among Children Who Were Seropositive at the Beginning of the Study Period

Seroreversion	Children Seropositive at Beginning of Study Period, No.		
	Jinja	Tororo	Total
Seroreversion event observed during study period			
Yes	37	52	89
No	161	70	231
Total	198	122	320

In addition, we compared half-lives of tetanus antitoxin IgG antibody concentrations using additional metrics of malaria exposure. We assessed half-lives of tetanus antitoxin IgG antibody concentrations among children across both study sites with HbS genotyping results available (n = 565/597); there was no significant difference in tetanus antitoxin IgG antibody concentration half-life between children with the HbAA genotype (n = 417; 4.2 [95% CI, 3.6–4.9] years) and those with the HbAS genotype (n = 144; 4.0 [3.3–4.8] years); there were not enough children with the HbSS genotype for inclusion in this comparison (n = 4). We also compared tetanus antitoxin IgG antibody decay among different groups of children by individual annual febrile malaria incidence and the proportion of months with any parasitemia across both study sites (Table 3 and Supplementary Figures 4 and 5). There were significant differences in tetanus antitoxin IgG antibody concentration half-lives among children in the lowest observed quartiles of individual annual febrile malaria incidence compared with those in higher observed quartiles; this trend was also observed among the 2 lowest quartiles of proportion of months with any parasitemia compared with the 2 higher quartiles.

**Table 3.** Tetanus Antitoxin Immunoglobulin G Antibody Decay Among Quartiles of Malaria Exposure Metrics (Both Study Sites)

Quartile	Individual Annual Febrile Malaria Incidence			Individual Proportion of Months With Any Parasitemia		
	Value Range, episodes per person-year	Children, No.	Half-Life (95% CI), y	Value Range, %	Children, No.	Half-Life (95% CI), y
Q1	0–0	177	13.5 (7.4–24.6)	0–3.3	150	22.6 (7.5–68.0)
Q2	0–0.7	122	5.4 (4.3–6.8)	3.3–19.4	149	6.2 (4.7–8.2)
Q3	0.7–2.3	149	3.2 (2.8–3.7)	19.4–40.0	150	3.4 (3.0–3.9)
Q4	2.3–9.6	149	2.6 (2.3–2.9)	40.0–88.6	148	2.7 (2.5–3.0)

Abbreviations: CI, confidence interval; Q1 (etc), quartile 1 (etc).

In a sensitivity analysis to further examine the association of decay among children living in the high malaria transmission district (ie, with the highest malaria exposure history) and remove comparisons across study sites, similar comparisons of individual annual febrile malaria incidence and proportion of months with any parasitemia among children living in Tororo only (Supplementary Table and Supplementary Figures 5 and 6) did not yield significant results. In addition, as data were available, we also compared tetanus antitoxin IgG antibody concentration half-lives among children across both study sites by groups of average individual hazard of malaria infection and household aEIR. A dose-response relationship was observed between quantiles of both indicators and tetanus antitoxin IgG antibody concentration half-lives (Supplementary Table 5 and Supplementary Figures 8 and 9).

## DISCUSSION

Vaccination against tetanus guards against infection and has helped facilitate progress toward maintaining and achieving maternal and neonatal tetanus elimination in many countries. However, vaccine-induced protection against tetanus is known to wane [36, 37], and, as such, additional booster doses are recommended to increase immunity [17]. Fewer opportunities to receive childhood vaccines exist in sub-Saharan Africa compared with high-income settings due to differences in access, supply, and immunization schedules. The tetanus burden is concentrated in settings of high malaria burden where suboptimal vaccination coverage persists [38] and tetanus antitoxin IgG antibody kinetics data are limited. Understanding the potential effects of malaria infections on tetanus antitoxin IgG antibody kinetics could provide actionable insights on vaccination strategies, particularly as countries consider introducing tetanus-containing boosters. In addition, population-level tetanus seroprevalence is often used as a marker of vaccination program performance [39, 40]; however, without considering waning, these estimates would be biased. We found in our study sites in Uganda, which do not deliver tetanus-containing booster doses for children prior to antenatal care visits, the estimated average age at seroreversion among children was 8.1 years.

In highly malaria-endemic settings, individuals may be infected hundreds of times per year [23, 41]. Malaria infections elicit short-term effects on host immunity (eg, altering phenotypes of the memory B-cell compartment [16, 42], increasing inflammatory cytokines [42]) and cause both widespread immune activation and regulatory responses [5]. Thus, an incident malaria episode could have transient or permanent effects on other antibody responses for unknown durations and magnitudes, and the cumulation of malaria infections over time could have an effect on other antibody responses [43].

Tetanus is a prime example for examining the effects of malaria exposure history on vaccine responses, as IgG antibodies against tetanus are exclusively generated via vaccination [17]. Multiple studies [44] have measured the impact of malaria infection or chemoprevention regimens on tetanus (and other) vaccine immunogenicity, with mixed results. To our knowledge, however, few studies have examined the possible implications of malaria on long-term VPD antibody concentrations; only 2 have examined these associations with tetanus vaccine. One study found nonsignificant associations in children between tetanus serostatus and a positive malaria blood smear at the time of sample collection [45], but that study did not adjust for age and considered only current malaria status. Another study also found no change in tetanus antitoxin IgG antibody concentrations in children before and after a malaria season [46], but that study did not explicitly examine this relationship by age or use quantifications of individual-level infection history during the malaria season to assess changes in concentrations. In our study, which was able to examine malaria exposure history and the long-term durability of tetanus antitoxin IgG antibody concentrations more robustly and dynamically, we found significantly faster rates of tetanus antitoxin IgG antibody decay among children living in Tororo district than among those living in Jinja district. While this finding is ecological, a main distinction between these districts that may be contributing to differences in decay rates is the higher malaria transmission in Tororo district.

However, when using different approaches to assessing malaria exposure histories in individuals, we observed variably significant effects on tetanus antitoxin IgG antibody decay. Significant results are likely related to differences in the annual febrile incidence and the proportion of months with any parasitemia by study site (ie, the lowest quartiles were made up exclusively by children living in Jinja district). This may be attributable in part to the challenges of quantifying malaria exposure, such as the complex natural history from febrile malaria episodes (ie, treated episodes with parasites cleared) versus chronic asymptomatic parasitemia (ie, not treated), and limitations that arise from grouping classifications of exposure, such as binning children into groups representing months of parasitemia, and small sample sizes (ie, HbS genotyping). We observed few children in Tororo with no history of malaria exposure. If having any malaria episode irreversibly affects the decay and variation in tetanus antitoxin IgG antibody concentrations, we would not be able to disentangle this difference among a cohort in Tororo without many children with no malaria history during the study period.

This study was not designed to test the impact of a single incident malaria episode on tetanus antitoxin IgG antibody kinetics, due to variable timing and the numbers of samples available per individual across differential time intervals. Future work is needed that directly considers effects of single malaria episodes on tetanus antitoxin IgG antibody concentrations, including baseline preinfection samples and postinfection samples at various time points.

Our analysis has multiple limitations. Vaccination history (ie, vaccination status or doses received) was not collected from study participants. Therefore, we were unable to conduct analyses relative to time since vaccination and assumed that age was an appropriate proxy. We cannot exhaustively exclude the possibility that children were not given booster doses under any circumstance (eg, injury or accident). Relatedly, while no such immunization campaign occurred in Tororo or Jinja during the study period, we are unable to verify that children received no other vaccinations that would consequently boost tetanus immunity (eg, MenAfriVac or other conjugate vaccines containing tetanus components). In addition, among additional indicators investigated (eg, stunting), there are confounding and complex relationships with malaria [35] for which we were unable to control. For example, it is challenging to determine whether malaria caused nutritional deficiencies or nutritional deficiencies lead to malaria susceptibility and infection; in addition, both nutritional deficiencies and malaria susceptibility may also be related to factors like socioeconomic status and thus linked by another related, yet undefined, mechanism. Finally, we were able to detect incident febrile malaria episodes or history of parasitemia only within the study period. There may be long-lasting effects of malaria exposure on tetanus antitoxin IgG antibody decay from previous occurrences, but we were not able to capture and quantify these exposures.

Our work suggests that to protect vulnerable children against tetanus infection, booster doses need to be introduced in all countries’ routine immunization schedules, including in Uganda where our study was conducted. Children with a history of malaria may be at a higher risk of earlier seroreversion; therefore, it may be important to consider local malaria transmission patterns to determine the optimal delivery timing of tetanus booster doses. Additional future work [44, 47] is needed to further explore the effect of malaria on antibodies against tetanus and other VPDs, with both fast and slow expected antibody waning (eg, pertussis, measles), to better understand susceptibility to infection and prevent avoidable disease burden and deaths.

## Supplementary Material

jiag255_Supplementary_Data

## Supplementary Data

Supplementary materials are available at *The Journal of Infectious Diseases* online (http://jid.oxfordjournals.org/). Supplementary materials consist of data provided by the author that are published to benefit the reader. The posted materials are not copyedited. The contents of all supplementary data are the sole responsibility of the authors. Questions or messages regarding errors should be addressed to the author.
