# Supplementary material for: Assessing the Effect of Malaria Exposure History on Tetanus Antibody Waning Rates Among Children in Jinja and Tororo Districts, Uganda
Source: J Infect Dis. 2026 May 9;234(4):e755–64. doi: 10.1093/infdis/jiag255 (PMC13668354; doi:10.1093/infdis/jiag255)
Supplement: Supplementary file 1 [file jiag255_supplementary_data.docx]

**Assessing the effect of malaria exposure history on tetanus antibody waning rates among children in Jinja and Tororo Districts, Uganda**

Supplementary Data

**Figure S1. Conversion from tetanus antitoxin MFI to IU/mL.**

Overview of conversion process from observed tetanus antitoxin MFI to IU/mL via an intermediate analysis of positive pooled hyperimmune serum from adults in a highly malaria endemic setting (i.e., CP3) from an additional study[1].

**Figure S2. Rug plot of samples and standards.**

For each plate run by site, standard curves of CP3 concentrations and tetanus antitoxin MFI are presented along with sample tetanus antitoxin MFI and CP3 concentration.

**Figure S3. Kaplan-Meier plot of probability of remaining seropositive by age across study sites.**

Corresponding plot to hazard model analysis suggesting significantly higher hazard of sero-reversion among children living in Tororo than children living in Jinja.

**Figure S4. Tetanus antitoxin IgG antibody concentrations (IU/mL)** **by age across quartiles of individual annual febrile malaria incidence.**

Tetanus antitoxin IgG antibody concentrations (IU/mL) by age across quartiles of individual annual malaria incidence, with the first (i.e., lowest) quartile in pink, the second quartile in orange, the third quartile in green, and the fourth quartile in blue. Dashed horizonal line indicates sero-protective threshold of 0.1 IU/mL.

**Figure S5. Tetanus antitoxin IgG antibody concentrations (IU/mL)** **by age across quartiles of individual proportion of months with any parasitemia.**

Tetanus antitoxin IgG antibody concentrations (IU/mL) by age across quartiles of proportion of months with any parasitemia, with the first (i.e., lowest) quartile in pink, the second quartile in orange, the third quartile in green, and the fourth quartile in blue. Dashed horizonal line indicates sero-protective threshold of 0.1 IU/mL.

**Figure S6. Tetanus antitoxin IgG antibody concentrations (IU/mL)** **by age across quartiles of individual annual febrile malaria incidence among children living in Tororo district.**

Tetanus antitoxin IgG antibody concentrations (IU/mL) by age across quartiles of individual annual malaria incidence, with the first (i.e., lowest) quartile in pink, the second quartile in orange, the third quartile in green, and the fourth quartile in blue. Dashed horizonal line indicates sero-protective threshold of 0.1 IU/mL.

**Figure S7. Tetanus antitoxin IgG antibody concentrations (IU/mL)** **by age across quartiles of individual proportion of months with any parasitemia among children living in Tororo district.**

Tetanus antitoxin IgG antibody concentrations (IU/mL) decay by age across quartiles of proportion of months with any parasitemia, with the first (i.e., lowest) quartile in pink, the second quartile in orange, the third quartile in green, and the fourth quartile in blue. Dashed horizonal line indicates sero-protective threshold of 0.1 IU/mL.

**Figure S8. Tetanus antitoxin IgG antibody concentrations (IU/mL)** **by age across quartiles of average individual hazard of malaria infection.**

Tetanus antitoxin IgG antibody concentrations (IU/mL) by age across quartiles of average individual hazard of malaria infection, with the first (i.e., lowest) quartile in pink, the second quartile in orange, the third quartile in green, and the fourth quartile in blue. Dashed horizonal line indicates sero-protective threshold of 0.1 IU/mL.

**Figure S9. Tetanus antitoxin IgG antibody concentrations (IU/mL)** **by age across quartiles of household annual entomological inoculation rate (aEIR).**

Tetanus antitoxin IgG antibody concentrations (IU/mL) by age across quartiles of household annual entomological inoculation rate (aEIR), with the first (i.e., lowest) quartile in pink, the second quartile in orange, the third quartile in green, and the fourth quartile in blue. Dashed horizonal line indicates sero-protective threshold of 0.1 IU/mL.

**Table S1. Model comparisons for model structure selection.**

Fit metrics including Akaike information criterion (AIC) and log-likelihood for each model tested are provided. Bolded models had the best overall fit. Based on most models selecting log-linear structures across combinations of study sites, log-linear decay models are used throughout the analysis. Number of model parameters includes fixed effects, variance component of individual-level random intercept, and residual variance (i.e., not every random intercept).

| Study sites | Number of individuals | Number of observations | Number of parameters | Structure | AIC | Log-likelihood |
| --- | --- | --- | --- | --- | --- | --- |
| Jinja only | 271 | 1650 | 4 | Log-linear | 3982 | -1987 |
|  |  |  |  | **Log-log** | **3975** | **-1984** |
| Tororo only | 326 | 1419 | 4 | **Log-linear** | **2791** | **-1391** |
|  |  |  |  | Log-log | 2835 | -1413 |
| Both Jinja and Tororo– sites combined (i.e., not stratified) | 597 | 3069 | 4 | **Log-linear** | **7185** | **-3589** |
|  |  |  |  | Log-log | 7224 | -3608 |
| Both Jinja and Tororo – sites stratified | 597 | 3069 | **5** | **Log-linear** | **6937** | **-3463** |
|  |  |  |  | Log-log | 6952 | -3471 |

**Table S2. Tetanus antitoxin IgG antibody concentration half-lives among children who were ever or never stunted or wasted in the study period living Tororo and Jinja districts.**

|  |  | Jinja | Tororo |
| --- | --- | --- | --- |
| Stunting status | Ever stunted | 5.3 years  (1.3 to 9.4 years) | 2.7 years  (2.2 to 3.3 years) |
|  | Never stunted | 16.7 years  (6.3 to 27.1 years) | 2.8 years  (2.6 to 3.1 years) |
| Wasting status | Ever wasted | 6.3 years  (3.3 to 9.3 years) | 2.3 years  (1.9 to 2.8 years) |
|  | Never wasted | 7.5 years  (2.5 to 12.4 years) | 2.0 years  (1.7 to 2.3 years) |

**Table S3. Sero-reversion by study site among individuals ever sero-positive during study period.**

Among individuals that were ever sero-positive during the study period, individuals with sero-reversion events observed by study site.

|  | Jinja | Tororo | Total |
| --- | --- | --- | --- |
| Sero-reversion event observed during study period | 67 | 71 | 138 |
| No sero-reversion event observed during study period | 172 | 73 | 245 |
| Total | 239 | 144 | 383 |

**Table S4. Tetanus antitoxin IgG antibody concentration half-lives among quartiles of malaria exposure metrics among children living in Tororo district.**

Individual annual febrile malaria incidence, and individual proportion of months with any parasitemia among children living in Tororo district.

|  | Individual annual febrile malaria incidence | | | Individual proportion of months with any parasitemia | | |
| --- | --- | --- | --- | --- | --- | --- |
|  | Value range | n | Half-life (95% CI) | Value range | n | Half-life (95% CI) |
| First quartile | 0 – 1.1 | 82 | 3.0 years  (2.6 – 3.5 years) | 0% – 24.0% | 82 | 2.7 years  (2.3 – 3.1 years) |
| Second quartile | 1.1 – 2.1 | 81 | 2.9 years  (2.5 – 3.4 years) | 24.0% – 37.4% | 81 | 3.1 years  (2.7 – 3.5 years) |
| Third quartile | 2.1 – 3.6 | 81 | 2.7 years  (2.4 – 3.1 years) | 37.4% – 50.0% | 93 | 2.6 years  (2.3 – 2.9 years) |
| Fourth quartile | 3.6 – 9.6 | 82 | 2.4 years  (2.2 – 2.8 years) | 50.0% – 88.6% | 70 | 2.8 years  (2.4 – 3.2 years) |

**Table S5. Tetanus antitoxin IgG antibody concentration half-lives among additional quartiles of malaria exposure metrics.**

Average individual hazard of malaria infection (n=292) and household annual entomological inoculation rates (aEIR) (n=409) among children living in both study sites where hazard and aEIR were available[2].

|  | Average individual hazard of malaria infection | | | Household annual entomological inoculation rate (aEIR) | | |
| --- | --- | --- | --- | --- | --- | --- |
|  | Value range | n | Half-life (95% CI) | Value range | n | Half-life (95% CI) |
| First quartile | 0.004 – 0.011 | 73 | 4.9 years  (3.8 – 6.5 years) | 1.5 –3.7 | 104 | 20.6 years  (7.4 – 57.5 years) |
| Second quartile | 0.011 – 0.015 | 73 | 3.0 years  (2.6 – 3.5 years) | 3.7 – 36.7 | 104 | 3.4 years  (2.9 – 4.0 years) |
| Third quartile | 0.015 – 0.020 | 73 | 3.1  (2.6 – 3.7 years) | 36.7 – 63.4 | 99 | 3.1 years  (2.7 – 3.5 years) |
| Fourth quartile | 0.020 – 0.041 | 73 | 2.5  (2.1 – 2.9 years) | 63.4 – 181.0 | 102 | 2.9 years  (2.5 – 3.3 years) |

**References**

1.	Carcelen AC, Monjane C, Bérubé S, et al. Multiplex bead assays enable integrated serological surveillance and reveal cross-pathogen vulnerabilities in Zambezia Province, Mozambique. Nat Commun. Nature Publishing Group; **2025**; 16(1):7946.

2.	Rodriguez-Barraquer I, Arinaitwe E, Jagannathan P, et al. Quantification of anti-parasite and anti-disease immunity to malaria as a function of age and exposure. Cooper B, Jha P, editors. eLife. eLife Sciences Publications, Ltd; **2018**; 7:e35832.
